# PD-L1^+^ macrophages are associated with favorable features in primary mediastinal (thymic) large B-cell lymphoma

**DOI:** 10.1186/s40164-023-00396-0

**Published:** 2023-03-20

**Authors:** Raphael E. Steiner, Edwin R. Parra, Francisco Vega, Lei Feng, Jason R. Westin, Sattva S. Neelapu, Paolo Strati, Michael R. Green, Christopher R. Flowers, Luisa M. Solis, Ignacio I. Wistuba, Sairah Ahmed, Ranjit Nair, Fredrick B. Hagemeister, Mansoor Noorani, Mario L. Marques-Piubelli

**Affiliations:** 1 Lymphoma and Myeloma, MD The University of Texas MD Anderson Cancer Center, 1515 Holcombe Blvd, Unit 429, Houston, TX 77030 USA; 2 Translational Molecular Pathology, MD Anderson Cancer Center, Houston, USA; 3 Hematophathology, MD Anderson Cancer Center, Houston, USA; 4 Biostatistics, MD Anderson Cancer Center, Houston, USA

**Keywords:** Primary mediastinal large B-cell lymphoma, Macrophages, PD-L1, CD30, Biomarker

## Abstract

Primary mediastinal (thymic) large B-cell lymphoma (PMBCL) is a rare, aggressive subtype of non-Hodgkin lymphoma and has a complex inflammatory microenvironment. Although most patients can be cured with standard-of-care immunochemotherapy, patients who have disease relapse have an unfavorable prognosis. Pre-treatment prognostic biomarkers in PMBCL are needed. In this retrospective study, we analyzed the clinical features and outcomes of PMBCL patients and their association with immune cell subpopulations identified by multiplex immunofluorescence at initial diagnosis. Two different antibody panels were used to assess macrophages in tissue biopsy specimens collected before the initiation of induction therapy. Twelve PMBCL patients, including five patients who had disease relapse, were included in the analysis. At a median follow-up time of 32.2 months, the median progression-free and overall survival durations were not reached. Our findings suggest that a high density of PD-L1^+^ macrophages is associated with favorable features, such as early disease stage and the absence of B-symptoms, and indicate that a high percentage of PD-L1^+^ macrophages and high densities of CD30^+^PD-L1^+^ cells and CD30^+^ cells might be associated with a lower risk of relapse within 12 months of therapy initiation. Further studies are needed to develop a biomarker signature predictive of treatment response with therapeutic consequences for patients with newly diagnosed PMBCL.

**Supplementary Information:**

The online version contains supplementary material available at 10.1186/s40164-023-00396-0.

To the editor

Primary mediastinal (thymic) large B-cell lymphoma (PMBCL) shares several features with classic Hodgkin lymphoma, such as 9p24.1 amplification, increased programmed cell death protein 1 (PD-1) ligand and CD30 expression [1, 2]. Between 7 and 20% of PMBCL patients have relapse after frontline chemoimmunotherapy, and most have dismal outcomes in spite of intensive therapy [3–5]. Predictive studies are needed to identify PMBCL patients with inferior outcomes, who may benefit from novel therapies or enrollment in a clinical trial.

In this retrospective study, we used multiplex immunofluorescence (mIF) to analyze pretreatment samples from PMBCL patients to identify prognostic characteristics and features associated with treatment response. MIF was not previously used to analyze PMBCL samples and enables better characterization of the cellular composition of the tumor microenvironment in terms of cellular subtypes and has increased sensitivity compared to conventional immunohistochemistry [6]. Methods are described in Additional file 1.

Patient and treatment characteristics are given in Table 1. Twelve PMBCL patients were included in this study. The median age at the time of initial diagnosis was 33 years (range, 22–54 years). Of the 12 patients, 7 (58%) had bulky disease, 5 (42%) had stage I or II disease, 7 (58%) had no B-symptoms, and 8 (67%) had an IPI score of 0 or 1. Nine patients (75%) received 6 cycles of dose-adjusted rituximab, etoposide, prednisone, vincristine, cyclophosphamide, and doxorubicin [4]), as a first line of therapy. However, no patients received consolidative radiotherapy in first complete remission. Five patients had disease relapse at a median of 6 months (range, 3–8 months) and received different additional therapies. At a median follow-up time of 32.2 months (range, 18.3–65.2 months), the median PFS and OS durations were not reached. At the most recent follow-up, one patient had died of progressive disease.

**Table 1:** Patient and treatment characteristics

Deidentified number	Gender	Race	Ethnicity	Age at diagnosis	B-SX at DX	Initial stage	IPI	Bulky > 10 cm	1st line of therapy	At least one relapse	2nd line of therapy	3rd line of therapy	4th line of therapy	FU in months	Status at last FU
PML01	Male	white	not Hispanic	46	No	II	1	Yes	6xDA-R-EPOCH	No				18	Alive
PML02	Female	Asian	not Hispanic	30	No	II	1	Yes	6xDA-R-EPOCH	No				32	Alive
PML03	Male	white	Hispanic	36	No	IV	2	No	6xDA-R-EPOCH	No				21	Alive
PML04	Male	white	not Hispanic	54	No	IV	2	Yes	6xDA-R-EPOCH	Yes	3xR-ICE, ASCT and XRT			65	Alive
PML05	Male	American Indian	not Hispanic	38	No	I	1	Yes	6xDA-R-EPOCH	No				26	Alive
PML06	Female	Hawaiian/Pacific Islander	Hispanic	29	Yes	II	1	Yes	6xDA-R-EPOCH	Yes	1 × R-DHAP	1xR-ICE, Liso-cel		29	Alive
PML07	Male	white	NA	30	Yes	III	1	Yes	1xABVD + 6xDA-R-EPOCH	Yes	3xR-DHAP, then ASCT	Selinexor and Rituximab	Rituximab + fractionated Cyclophosphamide	15	Dead
PML08	Female	white	not Hispanic	24	Yes	IV	2	No	6xDA-R-EPOCH	No				38	Alive
PML09	Female	white	not Hispanic	50	Yes	IV	3	Yes	6xDA-R-EPOCH	Yes	1xR-DHAP	Tisa-cel	7xPembrolizumab + XRT	31	Alive
PML10	Male	white	not Hispanic	22	No	II	0	No	4xDA-R-EPOCH	No				35	Alive
PML11	Male	white	not Hispanic	38	Yes	III	1	No	1xR-CHOP + Len, + 5xDA-R-EPOCH	No				37	Alive
PML12	Female	white	not Hispanic	28	No	IV	1	No	5xDA-R-EPOCH	Yes	Axi-cel	8xPembrolizumab, ASCT and XRT		57	Alive

ABVD: doxorubicin hydrochloride, bleomycin sulfate, vinblastine sulfate, and dacarbazine; ASCT: autologous stem cell transplantation; axi-cel: axicabtagene ciloleucel; DA-R-EPOCH: dose-adjusted rituximab, etoposide, prednisone, vincristine, cyclophosphamide, doxorubicin; DX: diagnosis; FU: follow-up; IPI: International Prognostic Index; Len: lenalidomide; Liso-cel: lisocabtagene maraleucel; NA: not available; R-DHAP: rituximab, cytosine arabinoside, dexamethasone; R-ICE: rituximab, ifosfamide, carboplatin, and etoposide; SX: symptoms;Tisa-cel: *tisagenlecleucel;* x: cycles; XRT: radiotherapy of the mediastinum

Per patient, the mean tissue area analyzed was 93.66 mm^2^ (range, 11.6–240.0 mm^2^) and the mean number of cells analyzed was 9658 cells (range, 6453–12,351 cells). The presented combination of markers output (Additional file 1: Table 1) is based on the previously most commonly assessed combinations [7–9].

Compared with patients who had stage III or IV disease, patients who had stage I or II PMBCL had a significantly higher mean density of CD68^+^PD-L1^+^ cells (115.4 vs. 21.2 cells/mm^2^; p = 0.02; Fig. 1A, D). Compared with patients with B-symptoms, patients without B-symptoms had a significantly higher mean percentage of CD68^+^PD-L1^+^ cells (26.2% vs. 3.9%; p = 0.01; Fig. 1B). Compared with patients who had disease relapse within 12 months of therapy initiation, patients who did not have relapse within 12 months had a non statistically significant higher mean percentage of CD68^+^PD-L1^+^ macrophages (23.6% vs. 7.6%; p = 0.14; Fig. 1C), higher mean density of CD30^+^PD-L1^+^ cells (156.9 vs. 38.1 cells/mm^2^, p = 0.14; Fig. 1E, Additional file 1: Figure 1), and higher mean density of CD30^+^ cells (2,394.6 vs. 283.5 cells/mm^2^; p = 0.14; Fig. 1F).

**Fig. 1:**
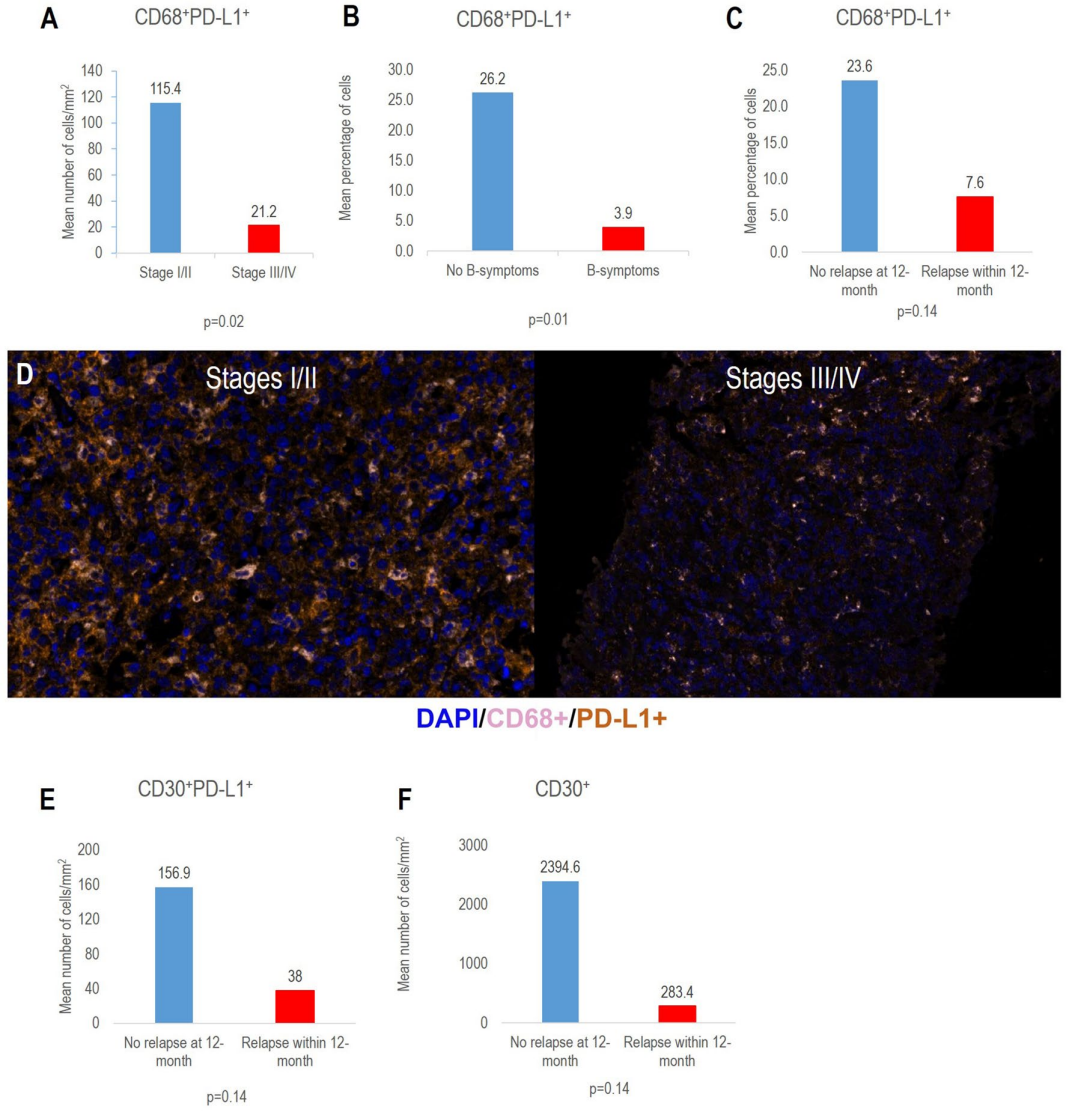
**A-F** Expression of PD-L1 and CD30 by multiplex immunofluorescence on pre-treatment biopsy and their association with clinical characteristics and outcome

Our results suggest that a high density and mean percentage of PD-L1^+^ macrophages in pre-treatment tissue biopsy samples is associated with favorable features, including early-stage disease and the absence of B-symptoms. Moreover, our results indicate that a high percentage of PD-L1^+^ macrophages or high densities of CD30^+^PD-L1^+^ cells or CD30^+^ cells in pre-treatment biopsy samples might be associated with a lower risk of relapse within 12 months of therapy initiation.

The present study had some limitations, including its small, single-center cohort and the lack of markers to precise the phenotype of lymphoma cells and further elements from the immune microenvironment. Further studies with additional patients are necessary to evaluate potential confounding factors such as early-stage disease and the absence of B-symptoms. Besides, additional research is warranted to develop PD-L1^+^ macrophages density and/or percentage as a potential response signature to predict response to checkpoint inhibitors in the frontline setting [10]. We also acknowledge the limitations of our study, including the need for a precise definition of the neoplastic cells since CD30 expression is negative in around 15% of cases, can have a heterogeneous pattern [2] and can be expressed in reactive B-cells of the background. A broader assessment using high-plex technologies, which allows the association of different markers to define specific cell phenotypes, is required to better understand the immune landscape of PMBCL [11]. Several clinical trials of PD-1 inhibitors with or without brentuximab vedotin as frontline therapy for PMBCL (notably NCT04745949 and NCT04759586) are ongoing [12]. The use of targeted therapies in the frontline setting may decrease chemoresistance. However, immune checkpoint inhibitors can cause immune-related adverse events and brentuximab vedotin can induce neurotoxicity and hematotoxicity. Predictive studies to improve the personalization of PMBCL patients and avoid the unnecessary use of certain therapies and their associated risks are an unmet need.

## Supplementary Information

**Additional file 1: Methods; Figure S1.** Representative pictures showing different cell densities of CD30+PD-L1+ cells. A-C) Multiplex immunofluorescence image of a case with low cell density of CD30+, PD-L1+, and CD30+PD-L1+ cells median: 20.46 cells/mm2, respectively. D-E) Multiplex immunofluorescence image of a case with high cell density of CD30+, PD-L1+, and CD30+PD-L1+ cells (median: 604.62 cells/mm2), respectively. Supplementary Table 1.

## Data Availability

The datasets used and/or analysed during the current study are available from the corresponding author on reasonable request.

## Abbreviations

IPI: International prognosis index scores
mIF: Multiplex immunofluorescence
OS: Overall survival
PFS: Progression-free survival
PMBCL: Primary mediastinal (thymic) large B-cell lymphoma
