# Supplementary material for: PD-L1+ macrophages are associated with favorable features in primary mediastinal (thymic) large B-cell lymphoma
Source: Exp Hematol Oncol. 2023 Mar 20;12:32. doi: 10.1186/s40164-023-00396-0 (PMC10026479; doi:10.1186/s40164-023-00396-0)
Supplement: Supplementary file 1 — Additional file 1: Methods; Figure S1. Representative pictures showing different cell densities of CD30+PD-L1+ cells. A-C) Multiplex immunofluorescence image of a case with low cell density of CD30+, PD-L1+, and CD30+PD-L1+ cells median: 20.46 cells/mm2, respectively. D-E) Multiplex immunofluorescence image of a case with high cell density of CD30+, PD-L1+, and CD30+PD-L1+ cells (median: 604.62 cells/mm2), respectively. Supplementary Table 1. [file 40164_2023_396_MOESM1_ESM.docx]

# Methods

This study was approved by MD Anderson’s Institutional Review Board and performed in accordance with the Declaration of Helsinki.

We searched the patient database of MD Anderson’s Department of Lymphoma and Myeloma to identify patients of age 18 years or older who had a clinicopathological diagnosis of PMBCL, who were treated at MD Anderson between April 2014 and November 2020, and for whom pre-treatment tissue samples were available. Clinical, radiological, and laboratory data, including International Prognosis Index scores (IPI),(1) were obtained from the patients’ medical records. Bulky disease was defined as a mass of at least 10 cm.

Multiplex immunofluorescence (mIF) was not previously used to analyze PMBCL samples and enables better characterization of the cellular composition of the tumor microenvironment in terms of cellular subtypes and their spatial relationship with the tumor cells and has increased sensitivity compared to conventional immunohistochemistry.(2)

Formalin-fixed, paraffin-embedded core biopsy tumor samples from the mediastinum were collected before induction therapy and used for this analysis. Two previously optimized and validated mIF panels were used for macrophage characterization. One panel included antibodies against CD68, CD137, PD-L1, PD-1, CD3, and CD30, and the other included antibodies against CD68, CD14, CD33, CD11b, arginase-1, and CD30. Each antibody was assessed with a uniplex immunofluorescence panel using the Opal 7-Color Manual IHC Kit (Akoya Biosciences, cat. no. NEL811001KT).(3, 4)

The slides were imaged with a Vectra spectral imaging system and high magnification (x20) were used to select all tumor areas in the biopsy. Healthy lymph node tissue samples were used as a positive control. After the preparation of the image, tissue was segmented in tumor and glass areas, followed by cell segmentation. Each marker was analyzed at the single-cell level, and a supervised algorithm was tailored for each case. Data consolidation was performed with the Spotfire software program (TIBCO, Palo Alto, CA).

Associations between categorical variables were evaluated using the chi-squared test or Fisher exact test. Differences in the immune cell densities and percentages of expression were assessed using Wilcox rank-sum test. Statistical software SAS 9.4 (SAS, Cary, NC) and S-Plus 8.2 (TIBCO Software Inc., Palo Alto, CA) were used for all the analyses.

Progression-free survival (PFS) was defined as the time from the date of therapy initiation to the date of disease progression or death, and overall survival (OS) was defined as the time from the date of therapy initiation to the date of death or last follow-up. The Kaplan-Meier method was used to calculate OS and PFS. Statistical analyses were performed with SAS 9.4 (SAS, Cary, NC) and Spotfire S+ 8.2.0 (TIBCO).

# Additional figure 1.


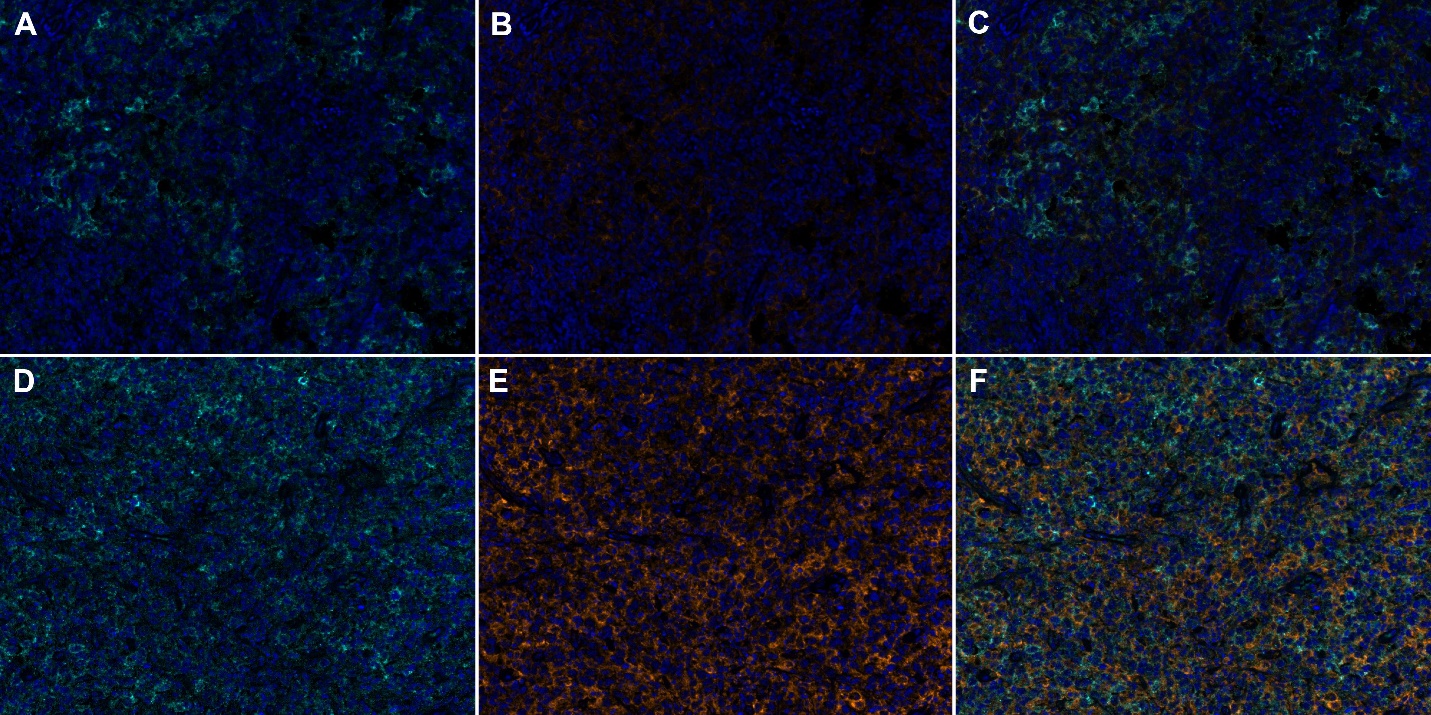


Representative pictures showing different cell densities of CD30+PD-L1+ cells. A-C) Multiplex immunofluorescence image of a case with low cell density of CD30+, PD-L1+, and CD30+PD-L1+ cells median: 20.46 cells/mm2, respectively. D-E) Multiplex immunofluorescence image of a case with high cell density of CD30+, PD-L1+, and CD30+PD-L1+ cells (median: 604.62 cells/mm2), respectively.

# Additional Table 1

| Case | Panel 1: CD3, CD30, CD68, CD137, PD-1, and PD-L1 | | | | | | | | | | | Panel 2: CD11b, CD14, CD30, CD33, CD68, and Arginase 1 | | | | | | |
| --- | --- | --- | --- | --- | --- | --- | --- | --- | --- | --- | --- | --- | --- | --- | --- | --- | --- | --- |
|  | CD30+ (cell/mm^2^) | CD30+PD-L1+ (cell/mm^2^) | CD3+ (cell/mm^2^) | CD3+PD-1+ (cell/mm^2^) | CD3+CD137+ (cell/mm^2^) | CD3+ CD137+PD-1+ (cell/mm^2^) | CD68+ (cell/mm^2^) | CD68+PD-L1+ (cell/mm^2^) | CD68+PD-1+ (cell/mm^2^) | CD68+CD137+ (cell/mm^2^) | CD30+CD137+PD-L1+ (cell/mm^2^) | CD30+ (cell/mm^2^) | CD68+ (cell/mm^2^) | CD68+ Arg1+ (cell/mm^2^) | CD68+CD11b+ (cell/mm^2^) | CD68+Arg1+CD11b+ (cell/mm^2^) | CD11b+Arg1+CD14+CD33+ (cell/mm^2^) | CD11b+CD33+ (cell/mm^2^) |
| PML01 | 244.76 | 65.83 | 2210.01 | 1714.51 | 23.34 | 21.54 | 575.69 | 260.32 | 176.00 | 54.46 | 28.13 | 454.13 | 467.76 | 0.62 | 199.50 | 0.62 | 0.00 | 0.62 |
| PML02 | 5294.43 | 44.67 | 919.00 | 764.55 | 7.66 | 7.02 | 327.39 | 62.54 | 60.00 | 18.51 | 1.28 | 1550.33 | 46.22 | 0.00 | 18.62 | 0.00 | 0.00 | 3.85 |
| PML03 | 363.93 | 7.00 | 592.55 | 490.68 | 20.22 | 20.22 | 73.10 | 10.89 | 8.00 | 0.00 | 0.00 | 228.62 | 156.89 | 0.00 | 0.75 | 0.00 | 0.00 | 0.00 |
| PML04 | 585.46 | 5.50 | 1597.90 | 92.44 | 180.48 | 20.91 | 209.09 | 42.92 | 2.00 | 4.40 | 1.10 | 867.23 | 145.29 | 0.00 | 0.00 | 0.00 | 0.00 | 0.00 |
| PML05 | 305.80 | 25.23 | 250.53 | 148.39 | 4.21 | 3.60 | 46.26 | 16.82 | 0.00 | 0.00 | 0.00 | 10.83 | 71.62 | 0.60 | 10.83 | 0.60 | 0.00 | 12.64 |
| PML06 | 21.62 | 4.20 | 2315.50 | 123.70 | 11.41 | 4.20 | 1007.63 | 126.70 | 38.00 | 3.60 | 0.60 | 247.11 | 479.90 | 0.00 | 5.97 | 0.00 | 0.60 | 35.81 |
| PML07 | 188.42 | 0.00 | 696.63 | 424.88 | 0.75 | 0.75 | 4.50 | 0.00 | 0.00 | 0.00 | 0.00 | 51.14 | 2.26 | 0.00 | 0.00 | 0.00 | 0.00 | 0.00 |
| PML08 | 5306.89 | 330.32 | 1043.45 | 588.94 | 7.68 | 7.68 | 82.58 | 1.28 | 2.00 | 0.00 | 0.00 | 4524.65 | 59.27 | 0.00 | 5.39 | 0.00 | 0.00 | 149.52 |
| PML09 | 353.96 | 37.36 | 2362.58 | 1831.03 | 7.35 | 5.51 | 988.39 | 23.88 | 113.00 | 2.45 | 0.61 | 1741.54 | 592.44 | 0.00 | 55.23 | 0.00 | 0.00 | 143.72 |
| PML10 | 4920.59 | 604.62 | 1080.19 | 68.11 | 11.95 | 0.60 | 247.34 | 110.53 | 2.00 | 3.58 | 1.79 | 5815.31 | 217.53 | 8.96 | 47.81 | 8.96 | 2.99 | 92.03 |
| PML11 | 325.58 | 20.46 | 4032.17 | 385.16 | 63.19 | 27.08 | 451.96 | 13.84 | 37.00 | 13.84 | 3.01 | 244.78 | 736.76 | 0.60 | 64.35 | 0.60 | 0.00 | 165.39 |
| PML12 | 267.74 | 143.11 | 2665.43 | 999.39 | 1.79 | 0.00 | 2139.50 | 55.46 | 180.00 | 1.19 | 0.60 | 728.69 | 333.70 | 0.00 | 117.10 | 0.00 | 0.61 | 319.14 |

# References

1. A predictive model for aggressive non-Hodgkin's lymphoma. The New England journal of medicine. 1993;329(14):987-94.

2. Tan WCC, Nerurkar SN, Cai HY, Ng HHM, Wu D, Wee YTF, et al. Overview of multiplex immunohistochemistry/immunofluorescence techniques in the era of cancer immunotherapy. Cancer Communications. 2020;40(4):135-53.

3. Zhang T, Liu H, Jiao L, Zhang Z, He J, Li L, et al. Genetic characteristics involving the PD-1/PD-L1/L2 and CD73/A2aR axes and the immunosuppressive microenvironment in DLBCL. Journal for immunotherapy of cancer. 2022;10(4).

4. Wang X, Zhang T, Song Z, Li L, Zhang X, Liu J, et al. Tumor CD73/A2aR adenosine immunosuppressive axis and tumor-infiltrating lymphocytes in diffuse large B-cell lymphoma: correlations with clinicopathological characteristics and clinical outcome. International journal of cancer. 2019;145(5):1414-22.
